# The prevalence, patterning and associations with depressive symptoms and self-rated health of emotional and economic intimate partner violence: a three-country population based study

**DOI:** 10.7189/jogh.10.010415

**Published:** 2020-06

**Authors:** Andrew Gibbs, Kristin Dunkle, Rachel Jewkes

**Affiliations:** 1 Gender and Health Research Unit, South African Medical Research Council, Pretoria, South Africa; 2 Centre for Rural Health, School of Nursing and Public Health, University of KwaZulu-Natal, Durban, South Africa; 3 School of Public Health, University of Witwatersrand, Johannesburg, South Africa; 4 Office of the President of the South African Medical Research Council, Cape Town, South Africa

## Abstract

**Background:**

Emotional and economic intimate partner violence (IPV) are common. There remain outstanding questions: 1) whether the patterning of emotional and economic IPV varies across contexts, and whether the current indicators adequately capture this variation; 2) whether simply binary or more complex modelling strategies are appropriate; 3) whether health impacts of emotional and economic IPV are sustained in population-based studies, across multiple settings.

**Methods:**

Ever partnered women (18-49 years) in cross-sectional, population-based data from three countries, China, Papua New Guinea (PNG) and Sri Lanka, from the United Nations Multi-country Study on Men and Violence in Asia and the Pacific. We assessed lifetime experience of emotional IPV (5 items) and economic IPV (4 items), item uniqueness (ie, the extent to which a person only reported that item), and descriptive associations and multivariable regression between combinations of emotional and economic IPV and physical and/or sexual IPV, for depressive symptoms and generalized health.

**Results:**

In all countries, lifetime emotional and economic IPV were common. By item, only one emotional IPV item (he hurt others of importance) had <3% of women uniquely identified by it. There was no item with low uniqueness for economic IPV. By item, and the entire scale, two or more experiences of emotional IPV, or economic IPV, were consistently associated with worse depression and generalized health. Emotional IPV was independently associated with higher depressive scores, and emotional IPV was independently associated with worse generalised health scores, across multiple models. Women experiencing physical and/or sexual IPV combined with emotional or economic IPV, reported the highest depressive symptoms and worst generalised health scores.

**Conclusions:**

Emotional IPV and economic IPV are more, or as, common as physical IPV and sexual IPV in three countries in Asia-Pacific. The current set of emotional and economic items captures a range of unique instances of IPV and that forms of emotional and economic IPV are patterned across different contexts. In addition, the use of a simple binary coding of these scales provides a robust way of providing a measure of health impact. The simplicity of this approach enables replication and standardization of measurement of these key constructs across multiple settings, enabling comparison.

Intimate partner violence (IPV) is a global public health problem [1]; the fifth Sustainable Development Goal includes a specific target and indicators to eliminate IPV [2]. While physical IPV and sexual IPV have received much attention, the scale and negative health impacts of women’s experiences of emotional and economic IPV are increasingly recognised [3]. Physical IPV refers to physically hurting a current or previous intimate partner, sexual IPV to coerced or physically forced sexual contact with a current or previous partner [4]. Emotional IPV includes insults and verbal abuse, as well as threats to do harm to a current or former partner or a member of their family, or pets (para-social violence) [5]. While economic IPV, refers to control of a current or former partner’s resources or seeking to limit their economic activities, and also includes throwing people out of their home, and not providing economic support to a family, when there are resources to do so [4].

Studies often show emotional IPV is the most common form of IPV women experience. For instance in an analysis of 12 Demographic Health Surveillance (DHS) surveys, from 10 African countries, lifetime emotional IPV ranged from 35.9% in Liberia, to 9.4% in Mali, and was typically the most common form of IPV [6]. However, population-based estimates of economic IPV experience are less common. In Australia a population-based sample estimated lifetime economic IPV to be 15.7% [7], while in Palestine, lifetime economic IPV was estimated to 45% [8]. Despite growing evidence about the frequency with which women experience emotional IPV, and economic IPV, there remains very little comparative population-based data from multiple countries, looking at all four forms of IPV in consistent ways.

Questions also remain as to whether emotional and economic IPV are patterned the same across different countries/contexts. In qualitative research on emotional IPV in Rwanda and South Africa, Stern et al [9] demonstrated that while there were differences in specific ways women experienced emotional IPV, linked to different norms around relationships and women’s mobility, the broad forms women experienced were remarkably similar. It is likely that women’s experiences of economic IPV are similarly varied, shaped by different work opportunities, norms about women’s work, and the levels of poverty. Understanding the patterning of individual indicators commonly used in emotional and economic IPV scales, may help us understand these differences, and whether there are any redundant items, which could be removed to reduce interviewee fatigue.

There is also evidence that emotional IPV and economic IPV have negative health impacts, independent from physical and sexual IPV. Studies have linked emotional IPV to post-natal depression [10], depressive symptomology [11,12] suicidal ideation [13-15], and psychosomatic conditions [12]. Similarly, despite less research, the negative mental health impacts of economic IPV have also been described, including higher depressive symptoms [8,11], suicidal ideation [11], and anxiety [8].

While evidence on the potential negative health impacts of emotional and economic IPV is growing, it remains limited. Much of the research on this topic has used small, non-representative samples and/or failed to account for co-occurrence of physical and/or sexual IPV. Thus, important questions remain regarding the extent to which emotional and economic IPV are independent risk factors for adverse health outcomes, as well as the extent to which their impact is obscured or potentially magnified when they co-occur with physical and/or sexual IPV. There is also not yet an agreed standard for the correct measurement of emotional or economic IPV, the items/constructs to be included and whether these phenomenon are best-modelled using simple binary approaches, or more complex scoring to demonstrate health impacts.

This paper draws on population-based data from three countries in the Asia-Pacific region to address four objectives. First, to estimate the prevalence of lifetime experience of emotional IPV and economic IPV in these populations. Second, to describe the lifetime prevalence of individual items within each scale, and assess their unique contribution to the scale, by country. Third, to assess whether particular items from a scale are more strongly associated with adverse health impacts. Fourth, to assess whether emotional IPV and economic IPV make independent contributions to self-reported health outcomes while accounting for co-occurrence of physical and/or sexual IPV.

## METHODS

The study was conceptualized by Partners for Prevention (P4P), with support from the South African Medical Research Council and country research teams. The overall P4P study was designed to assess men’s perpetration of IPV in six countries. Four of these sites also sampled women, and were considered for inclusion here. However only three of them included measures of health outcomes for female participants. Those were Sri Lanka, China and Papua New Guinea (PNG).

### Ethics

Ethical approval for the research was provided by the Medical Research Council of South Africa; the College of Humanities, Beijing Forestry University; and the Faculty of Medicine at the University of Colombo, Sri Lanka [16]. We followed ethical and safety guidelines for research on violence against women [4]. Interviewees received an information sheet and provided written consent.

### Sample

Data collection sites varied. In Sri Lanka, the city of Colombo and three contrasting districts were study sites. In China, research took place in a single county, with sampling split between a rural area and town. In PNG, there was a representative sample of the island of Bougainville. Further details of the research can be found elsewhere [16,17].

In each site, we selected census enumeration areas, with a probability proportionate to size, and systematically selected households within these areas. In households, we invited a woman aged 18–49 years (where necessary, randomly selected) for a face-to-face interview, with a trained female interviewer). In China, a household list of individuals in each cluster by age and sex was available and used for sampling within selected clusters, and the entire questionnaire was self-completed. Full details of the methods, sampling, are presented elsewhere [16,17]. The proportion of enumerated and eligible women interviewed per site varied, in Sri Lanka it was 73.9%, China 84.9%, and PNG 85.0% [16,17].

### Measures

#### Health outcomes

Two health outcomes were assessed across the countries, depression and self-rated health. In China and PNG depressive symptomology was assessed through ten items drawn from the Center for Epidemiologic Studies Depression (CESD) Scale [18]. Each item asked about clinically relevant symptoms of depression in the past week, with responses, ‘rarely or never, some or a little, moderate amount of time, most or all the time’. Scores were summed directly with higher scores indicating greater symptoms of depression (range 0-30; Cronbach α = 0.86 China, α = 0.89 PNG). Given that the CESD10 is a screening tool and not a method for diagnosing depression it was treated as a score in analyses, but for descriptive purposes a cut of 8 or more was used as more indicative of high levels of depressive symptomology [19]. The CESD was not asked in Sri Lanka, based on a decision by the country-team.

Self-rated generalised health was assessed in all three countries through a single item: “In general, would you describe your overall health as excellent, good, fair, poor or very poor?” Responses were on a five-point scale, from excellent to very poor. Higher scores indicated worse health.

### Intimate partner violence measures

The different forms of IPV women may have experienced were assessed through standardized measures in all three countries, based on questions first developed for the WHO multi-country survey on women’s health [3], and only asked to women who reported ever being partnered in their lifetime. These items have been refined in multiple studies globally and previously used in the Asia-Pacific context [3]. To assess emotional IPV, we asked women five questions about their lifetime experiences, for instance: “Has a current or previous husband or boyfriend ever insulted you or made you feel bad about yourself?” Responses were ‘never, once, few, or many times’. Similarly, four items asked about women’s lifetime experiences of economic IPV. An example question was: “Has a current or previous husband or boyfriend ever taken your earnings from you?” Responses were ‘never’, ‘once’, ‘few’, or ‘many times’. 

Lifetime experience of physical IPV and sexual IPV were assessed through five and four, behaviourally specific items, respectively. An example of physical IPV was: “Has a current or previous husband or boyfriend ever hit you with a fist or with something else which could hurt you?”, while for sexual IPV: “Has a current or previous husband or boyfriend ever physically forced you to have sex when you did not want to?” Response options for both scales were “never, once, few, or many times”.

All three countries included a series of standardized questions about socio-demographics, including age, highest level of education, current relationship status, food-security and ability to mobilise resources (cash) in an emergency. We also asked about nine experiences of childhood abuse and neglect, using items adapted from the Childhood Trauma Questionnaire.

### Analysis

The country data sets were combined into one. Data analysis were conducted using Stata IC/14.1 (StataCorp, College Station, TX, USA). Procedures accounted for the multistage structure of the data set, with stratification by data collection site within countries and enumeration areas as clusters. The data set was self-weighting.

First, we describe the sample by country, and as a pooled data set, using percentages and means as appropriate. We then examine the prevalence of each type of IPV assessed (emotional, economic, physical, sexual), through creating a three-level categorical variable to indicate lifetime experience of IPV: never, once, more than once. We then created an overall IPV experience variable, which parsed out all possible experiences of IPV, to look at overlaps and co-occurrence. This is also presented as a proportional Venn diagram. Descriptive analyses took into account the clustered nature of data, and 95% confidence intervals (CIs) were calculated using Taylor linearization [20], to account for data clustering.

Second, to describe prevalence and patterning of emotional IPV and economic IPV by item we: i) provide individual item responses as a percentage by country; and ii) estimate the item’s uniqueness, as the proportion of women who only reported that item in the scale. Essentially identifying the proportion that would not be categorised as experiencing that form of IPV if the item were not asked.

To assess associations between emotional IPV and economic IPV and health outcomes by individual items, and by country, we report the prevalence of each item by i) lifetime never/ever, ii) none or once, compared to few or more lifetime experiences, and iii) lifetime frequency of exposure (never, once, few, many). We present mean scores, and 95% CIs for depressive symptoms (China and PNG), and mean scores and 95% CIs for self-rated health (all countries). Where there is no overlap between 95% CIs, we consider this indication of the mean scores being different.

To assess whether emotional and economic IPV have independent associations with health outcomes, we pooled the data from all countries; country level analysis was infeasible because of sample size issues. We first assess this descriptively, through creating four-level categorical variables for multiple combinations of emotional and physical and/or sexual IPV, and economic and physical and/or sexual IPV. For instance, we created a four-level variable describing women who had i) no exposure to IPV ii) only exposure to economic IPV in their lifetime, iii) exposure to economic IPV and physical IPV in their lifetime, and iv) only exposure to physical IPV in their lifetime. We created a range of these four-level variables, based on frequency of exposure categories (never vs ever, and never or once, vs few or many) and types of exposure (sexual, physical, physical and/or sexual). For each permutation, we provide mean scores and 95% CI for depressive symptoms and self-reported health. We compare whether there is overlap between 95% CIs of each category and those reporting no IPV experience.

We then use unadjusted and adjusted multivariable regression models to assess whether IPV was independently associated with the health outcome in multiple ways. For depression, we used a negative binomial regression, and for self-rated health, ordered logistic regression. First, we separately model the impact of emotional, economic, and physical and/or sexual IPV, with no allowance for co-occurrence (Models 1-3), and then consider them together in a single model (Model 4). However, given that the constructs are largely overlapping, there was significant collinearity. We thus constructed four-level categorical variables comparing combinations of emotional IPV and physical and/or sexual IPV, and economic IPV and physical and/or sexual IPV experiences and health outcomes (Models 6-8). Finally, we created an eight level variable, which categorized women into all possible IPV combinations across the types of IPV its association with health (Models 9 and 10). All adjusted models included age, education, poverty and childhood traumas. All models included adjustment for the survey structure. We present the results of all of these models to explore the extent to which the measurement strategy impacts the point estimates for effect sizes and the conclusions drawn.

## RESULTS

### Description of the sample

In total 2438 ever partnered women were interviewed across the three countries (Sri Lanka n = 559; PNG n = 792; China n = 1087). In all countries (**Table 1**), the largest proportion of women were aged 35-49 years old (49.5% of the overall sample). In Sri Lanka and China over half the women interviewed (53.9% and 57.5% respectively) had some secondary education, but only 13.4% in PNG. Very few had no education whatsoever.

**Table 1:** Descriptive statistics and intimate partner violence experience per country, and combined

	Sri Lanka		China		PNG		Combined	
	**n**	**%/mean (95% CI)**	**n**	**%/mean (95% CI)**	**n**	**%/mean(95% CI)**	**n**	**%/mean (95% CI)**
**Age (years):**								
18-24	96	17.2 (14.1-20.7)	122	11.3 (9.6-13.2)	165	20.8 (17.9-24.1)	383	15.7 (14.3-17.3)
25-34	214	38.3 (32.7-44.2)	328	30.3 (27.0-33.9)	303	38.3 (35.1-41.6)	845	34.7 (32.5-37.0)
35-49	249	44.5 (38.7-50.6)	632	58.4 (54.7-62.1)	324	40.9 (37.0-45.0)	1205	49.5 (47.0-52.1)
**Education:**								
None	35	6.3 (4.3-9.1)	10	0.9 (0.5-1.8)	47	5.9 (4.4-8.0)	92	3.8 (3.0-4.8)
Incomplete primary	23	4.1 (2.5-6.7)	98	9.1 (7.1-11.5)	205	25.9 (22.6-29.4)	326	13.4 (11.6-15.4)
Complete primary	35	6.3 (4.0-9.6)	153	14.1 (11.9-16.7)	208	26.3 (22.9-29.9)	396	16.3 (14.4-18.3)
Incomplete secondary	301	53.9 (48.1-59.6)	622	57.5 (54.3-60.6)	106	13.4 (10.7-16.7)	1029	42.3 (39.3-45.4)
Complete secondary/higher	164	29.4 (24.3-35.1)	199	18.4 (15.9-21.1)	226	28.5 (24.7-32.7)	589	24.2 (22.2-26.4)
Relationship status:								
Married	503	90.0 (86.8-92.5)	1004	92.8 (91.0-94.2)	608	76.8 (73.3-79.9)	2115	86.9 (85.2-88.5)
**Intimate partner violence experience:**								
Ever emotional IPV:								
No experience	366	70.5 (66.0-74.7)	631	62.3 (58.9-65.5)	243	31.0 (27.8-34.4)	1240	53.5 (51.0-56.0)
Once	30	5.8 (4.1-8.1)	129	12.7 (10.8-14.9)	35	4.5 (3.2-6.1)	194	8.4 (7.2-9.7)
Two or more	123	23.7 (20.1-27.7)	253	25.0 (22.2-28.0)	507	64.6 (60.8-68.2)	883	38.1 (35.5-40.8)
**Ever economic IPV:**								
No experience	373	73.0 (64.0-80.4)	755	75.3 (73.0-77.4)	350	44.6 (39.8-49.5)	1478	64.3 (61.4-67.1)
Once	24	4.7 (2.9-7.5)	73	7.3 (6.0-8.9)	44	5.6 (4.3-7.3)	141	6.1 (5.3-7.1)
Two or more	114	22.3 (15.2-31.6)	175	17.5 (15.5-19.6)	391	49.8 (44.7-54.9)	680	29.6 (26.8-32.6)
**Ever emotional and/or economic IPV:**								
No experience	309	61.3 (53.1-68.9)	535	53.7 (50.4-57.0)	170	21.7 (18.7-25.0)	1014	44.4 (41.7-47.2)
Once	32	6.3 (4.5-8.9)	125	12.6 (10.6-14.8)	34	4.3 (2.9-6.5)	191	8.4 (7.2-9.7)
Two or more	163	32.3 (25.0-40.7)	336	33.7 (30.8-36.9)	580	74.0 (70.1-77.6)	1079	47.2 (44.3-50.2)
**Ever physical IPV:**								
No experience	418	79.5 (75.5-82.9)	665	65.0 (61.4-68.5)	381	48.5 (44.1-53.0)	1464	62.7 (60.1-65.3)
Once	23	4.4 (3.0-6.4)	113	11.1 (9.0-13.5)	52	6.6 (5.0-8.6)	188	8.1 (6.9-9.4)
Two or more	85	16.2 (12.5-20.7)	245	24.0 (21.3-26.8)	352	44.8 (40.8-48.9)	682	29.2 (27.0-31.6)
**Ever sexual IPV:**								
No experience	426	83.0 (78.2-87.0)	859	86.0 (83.3-88.3)	328	41.8 (37.5-46.2)	1613	70.2 (67.2-73.1)
Once	17	3.3 (2.1-5.2)	60	6.0 (4.3-8.4)	25	3.2 (2.2-4.5)	102	4.4 (3.5-5.6)
Two or more	70	13.7 (10.2-18.0)	80	8.0 (6.5-9.8)	432	55.0 (50.7-59.3)	582	25.3 (22.6-28.3)
**Ever physical and/or sexual IPV:**								
No experience	363	71.6 (66.5-76.2)	589	59.3 (54.8-63.6)	249	31.8 (28.1-35.6)	1201	52.6 (49.6-55.5)
Once	28	5.5 (3.7-8.3)	125	12.6 (10.4-15.1)	35	4.5 (3.2-6.3)	188	8.2 (7.0-9.7)
Two or more	116	22.9 (18.4-28.1)	280	28.2 (25.1-31.4)	500	63.8 (59.9-67.5)	896	39.2 (36.5-42.0)
**All IPV experiences:**								
None	265	54.1 (46.7-61.3)	405	41.6 (37.6-45.8)	133	17.0 (14.1-20.3)	803	35.8 (33.1-38.5)
Emotional only	28	5.7 (3.6-9.0)	82	8.4 (7.0-10.1)	47	6.0 (4.6-7.9)	157	7.0 (6.0-8.1)
Economic only	35	7.1 (4.5-11.2)	57	5.9 (4.3-7.9)	42	5.4 (3.7-7.7)	134	6.0 (4.8-7.3)
Emotional and economic only	22	4.5 (2.1-9.2)	33	3.4 (2.4-4.7)	26	3.3 (2.2-4.9)	81	3.6 (2.7-4.7)
Emotional and physical/sexual only	29	5.9 (3.7-9.3)	128	13.2 (11.2-15.5)	132	16.9 (14.3-19.8)	289	12.9 (11.4-14.5)
Economic and physical/sexual only	12	2.4 (1.1-5.3)	24	2.5 (1.7-3.6)	30	3.8 (2.5-5.8)	66	2.9 (2.2-3.8)
Physical/sexual only	36	7.3 (4.8-11.1)	118	12.1 (10.0-14.6)	37	4.7 (3.3-6.7)	191	8.5 (7.2-10.0)
All forms of IPV	63	12.9 (9.9-16.5)	126	13.0 (11.2-14.9)	336	42.9 (38.5-37.5)	525	23.4 (21.2-25.8)
**Health outcomes:**								
Depression (mean)			982	4.4 (4.0-4.8)	780	8.1 (7.3-8.9)	1742	10.5 (10.0-11.1)
Depression (8 or more)			264	27.4 (24.4-30.7)	393	50.4 (44.4-56.4)	657	37.7 (34.4-41.2)
Self-perceived health (mean)	532	2.0 (1.8-2.2)	1031	2.0 (2.0-2.1)	782	2.5 (2.4-2.7)	2345	2.2 (2.1-2.3)

CI – confidence interval, PNG – Papau New Guinea, IPV – intimate partner violence

In all countries, lifetime emotional IPV was the most prevalent form of IPV women reported (**Table 1**), but with wide variation from 29.5% in Sri Lanka to 61.0% in PNG. Overwhelmingly, women who reported emotional IPV were likely to report either “few” or “many” episodes over their lifetime, rather than just “once”.

Lifetime economic IPV was reported by 35.7% of the overall sample, with variation by country (Sri Lanka 26.3%; China 24.8%; PNG 55.4% - **Table 1**). In Sri Lanka and PNG, economic IPV was as common as physical IPV, while in China it was less common than physical IPV, but more common than sexual IPV. Women who reported any exposure to economic IPV, mainly reported experiencing either “few” or “many” experiences, rather than just “once” in their lifetime.

**Figure 1:**
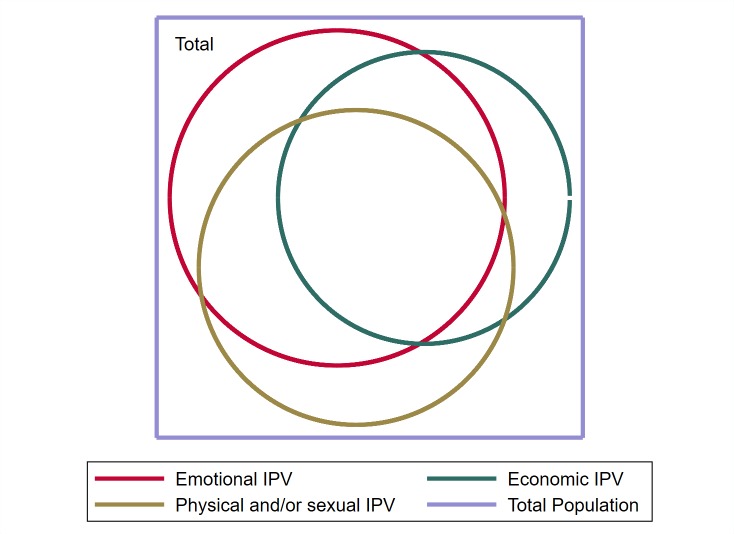
Proportional Venn diagram demonstrating overlaps between different forms of IPV women experienced in the three countries (pooled data).

When all forms of lifetime IPV were considered together and parsed out (**Table 1**) 35.8% reported never experiencing any IPV, 7.0% only emotional IPV, 6.0% only economic IPV, and 3.6% emotional and economic IPV only. A quarter (23.4%) reported lifetime experience of all forms of IPV. The Proportional Venn diagram (**Figure 1**) demonstrates these overlaps clearly.

### Variation in reporting of items by country

**Table 2** shows the prevalence of each individual item for emotional IPV. The most common emotional IPV item varied by country: in PNG it was ‘being insulted’ (55.4%), while in Sri Lanka and China it was ‘being scared or intimidated’ (22.1% and 23.8% respectively). In all countries, ‘hurt people you care about’ was the least reported item.

**Table 2:** Comparison by item and overall “coverage” and unique identification

Emotional IPV		Overall emotional violence % (n)	Item % (n)	Unique identification % (n)
Has a current or previous husband or boyfriend ever insulted you or made you feel bad about yourself?	Sri Lanka	29.5 (153)	14.8 (78)	4.6 (7)
	China	37.7 (382)	20.1 (206)	15.4 (59)
	PNG	69.0 (542)	55.2 (434)	13.3 (72)
Has a current or previous husband or boyfriend ever belittled or humiliated you in front of other people?	Sri Lanka	29.5 (153)	11.4 (60)	1.3 (2)
	China	37.7 (382)	15.3 (157)	5.2 (20)
	PNG	69.0 (542)	36.2 (284)	1.5 (8)
Has a current or previous husband or boyfriend ever done things to scare or intimidate you on purpose for example, by the way he looked at you, by yelling or smashing things?	Sri Lanka	29.5 (153)	22.1 (116)	24.2 (37)
	China	37.7 (382)	23.8 (244)	18.8 (72)
	PNG	69.0 (542)	42.9 (337)	3.0 (16)
Has a current or previous husband or boyfriend ever threatened to hurt you?	Sri Lanka	29.5 (153)	14.3 (75)	7.2 (11)
	China	37.7 (382)	11.0 (113)	3.4 (13)
	PNG	69.0 (542)	42.4 (333)	2.6 (14)
Has a current or previous husband or boyfriend ever hurt people you care about as a way of hurting you, or damaged things of importance to you?	Sri Lanka	29.5 (153)	10.6 (56)	2.6 (4)
	China	37.7 (382)	8.2 (84)	2.4 (9)
	PNG	69.0 (542)	26.4 (207)	2.2 (12)
**Economic IPV**		**Overall economic violence % (n)**	**Item % (n)**	**Unique identification % (n)**
Has a current or previous husband or boyfriend ever prohibited you from getting a job, going to work, trading, earning money or participating in income generation projects?	Sri Lanka	27.1 (138)	16.7 (88)	39.1 (54)
	China	24.7 (248)	14.4 (147)	43.5 (108)
	PNG	55.4 (435)	21.0 (165)	5.1 (22)
Has a current or previous husband or boyfriend ever taken your earnings from you?	Sri Lanka	27.1 (138)	13.0 (68)	21.0 (29)
	China	24.7 (248)	9.5 (97)	22.6 (56)
	PNG	55.4 (435)	35.0 (275)	22.5 (98)
Has a current or previous husband or boyfriend ever thrown you or your children out of the house where you were living?	Sri Lanka	27.1 (138)	2.5 (13)	0.7 (1)
	China	24.7 (248)	4.3 (44)	4.8 (12)
	PNG	55.4 (435)	21.9 (172)	6.0 (26)
Has a current or previous husband or boyfriend ever refused to give you money you needed for household expenses even when he has money for other things?	Sri Lanka	27.1 (138)	6.4 (33)	7.2 (10)
	China	24.7 (248)	4.7 (48)	5.2 (13)
	PNG	55.4 (435)	28.0 (220)	12.6 (55)

The extent to which women only reported that item for emotional IPV – uniqueness – varied by country. In all countries, the item “hurt others” was uniquely reported by <3% of participants, but there was no other item showing such consistency.

For economic IPV (**Table 2**) the most prevalent item varied across country; in PNG ‘earnings taken’ was reported by a third (35.0%) of women, while in Sri Lanka and China the most common form was “prohibited from getting a job” (16.7%; 14.4% respectively). In all three countries, the least common form of economic IPV reported was “thrown out of house” (2.5% Sri Lanka; 4.3% China; 21.9% PNG).

For economic IPV there was no clear patterning for uniqueness of responses by country. ‘Prohibited from working’ had high unique percentages in Sri Lanka and China (39.1% and 43.5% respectively), but not PNG (5.1%). While for a fifth of women in all three countries the only form of economic IPV they reported was ‘having earnings taken’ (21.0% Sri Lanka; 22.6% China; 22.5% PNG). Overall, the lowest unique item in all three countries was “thrown out of house” where uniqueness ranged from 0.7% (Sri Lanka) to 6.0% (PNG).

### Descriptive association between emotional IPV items and health outcomes

Descriptive associations between each emotional IPV scale item and health symptom mean scores by country were assessed (Table S1 in the **Online Supplementary Document**). For individual items of emotional IPV, there was a consistent patterning for the never/ever binary, and never or once vs ‘few or many’ binary, and their association with health impacts, with women reporting experiencing these forms of emotional IPV having higher means scores of depressive symptoms and worse self-reported health compared to those reporting no experience. For depressive symptomology, there was no overlap between 95% CI for any item, and for self-rated health there was no overlap between 95% CIs in 14/30 instances.

By the frequency of reported lifetime experience (never, once, few, many) for individual emotional IPV items (Table S1 in the **Online Supplementary Document**), there was a consistent gradation, whereby women reporting more experiences of any item, reported higher mean depressive symptoms. For all items of the emotional IPV scale, women reporting ‘few’ or ‘many’ experiences had higher mean depressive symptoms, with no overlap of 95% CIs, compared to those reporting “never”.

For self-reported health, frequency of experience by emotional IPV item was associated with worse health, compared to those reporting no experience (Table S1 in the **Online Supplementary Document**). Only with one item “scare or intimidate you” was there consistency across countries, whereby in all three countries those reporting ‘many’ experiences had no overlap with “never” for 95% CIs. For “insulted you” there was no overlap between 95% CIs for China and PNG, among women reporting “many” experiences of this, compared to “never”.

### Descriptive association between economic IPV items and health outcomes

Individual items of the economic IPV scale were assessed by health outcomes. For each item (Table S2 in the **Online Supplementary Document**), there was a similar patterning to emotional IPV, whereby higher mean scores were reported by those reporting more experiences of economic IPV. For depressive symptoms for never/ever, and never or once/two or more experiences, there was no overlap between 95% CIs for 13/16 combinations. For self-rated health, similarly the majority of binary combinations (17/24) had no overlap of 95% CIs.

For the frequency of experience of individual economic IPV items (Table S2 in the **Online Supplementary Document**), those reporting “once”, “few” or “many” experiences for each item had higher mean scores for depressive symptoms and self-rated health, compared to those reporting “never”. However, there was little consistency, whereby in 4/8 combinations for depressive symptoms women reporting ‘few’ had higher depressive scores than those reporting ‘many’ experiences.

### Descriptive and adjusted associations between different combinations of IPV and health outcomes

We also assessed descriptive (Table S3 in the **Online Supplementary Document**), unadjusted and adjusted associations (**Table 3****,** **Table 4** and **Table 5**) between different IPV combinations and health outcomes. For emotional IPV there are relatively consistent patterns. In all different models (descriptive and regression) emotional IPV is associated with more depressive symptoms, whether emotional IPV was coded as one or more experience, or two or more experiences. However, there is less clear patterning between emotional IPV and self-rated health; descriptively (Table S3 in the **Online Supplementary Document**) in 2/6 cases there is overlap between 95% CIs for emotional IPV only, and no IPV experience. In adjusted regressions in 3/6 models (Models 6, 9 and 10 in **Table 4** and **Table 5**) emotional IPV was not associated with self-rated health.

**Table 3:** Unadjusted and adjusted associations between IPV experiences and health outcomes*

	Depression†				Self-rated health‡			
	**Unadjusted**		**Adjusted§**		**Unadjusted**		Adjusted*	
	**β (95% CI)**	***P*-value**	**β (95% CI)**	***P*-value**	**β (95% CI)**	***P*-value**	**β (95% CI)**	***P*-value**
Model 1: Emotional IPV (1 or more)	0.63 (0.52, 0.73)	<0.001	0.37 (0.26, 0.47)	<0.001	0.80 (0.63, 0.97)	<0.001	0.47 (0.29, 0.66)	<0.001
Model 2: Economic IPV (1 or more)	0.53 (0.42, 0.65)	<0.001	0.27 (0.15, 0.38)	<0.001	0.75 (0.55, 0.94)	<0.001	0.45 (0.26, 0.65)	<0.001
Model 3: Physical and/or sexual IPV (1 or more)	0.63 (0.52, 0.74)	<0.001	0.37 (0.26, 0.48)	<0.001	0.75 (0.57, 0.93)	<0.001	0.44 (0.25, 0.63)	<0.001
Model 4:								
Emotional IPV (1 or more)	0.32 (0.19, 0.45)	<0.001	0.21 (0.08, 0.33)	0.001	0.44 (0.23, 0.65)	<0.001	0.27 (0.05, 0.50)	0.017
Economic IPV (1 or more)	0.26 (0.15, 0.37)	<0.001	0.12 (0.00, 0.23)	0.05	0.42 (0.18, 0.65)	0.001	0.28 (0.05, 0.51)	0.016
Physical and/or sexual IPV (1 or more)	0.36 (0.23, 0.48)	<0.001	0.24 (0.11, 0.37)	<0.001	0.36 (0.12, 0.59)	0.003	0.24 (0.01, 0.47)	0.045

*Models 1-3: Independent association between individual IPV predictors and health outcome (not adjusting for other IPV experiences); Model 4: Independent association between IPV experiences and health outcomes, adjusting for other IPV experiences.

†Negative binomial regression.

‡Ordered logistic regression.

§Adjusted for: age, education, poverty and childhood traumas.

**Table 4:** Unadjusted and adjusted associations between different IPV combinations and health outcomes*

	Depression†				Self-rated health‡			
	**Unadjusted**		**Adjusted§**		**Unadjusted**		**Adjusted§**	
	**β (95% CI)**	***P*-value**	**β (95% CI)**	***P*-value**	**β (95% CI)**	***P*-value**	**β (95% CI)**	***P*-value**
**Model 5: None**	**ref**		**ref**		**ref**		**ref**	
Emotional only (1 or more)	0.37 (0.20, 0.54)	<0.001	0.31 (0.15, 0.47)	<0.001	0.43 (0.15, 0.70)	0.003	0.28 (0.01, 0.55)	0.04
Emotional (1 or more) and p/s (1 or more)	0.81 (0.69, 0.93)	<0.001	0.50 (0.39, 0.62)	<0.001	1.00 (0.79, 1.20)	<0.001	0.61 (0.39 0.84)	<0.001
Physical and/or sexual only (1 or more)	0.39 (0.23, 0.55)	<0.001	0.32 (0.17, 0.48)	<0.001	0.32 (0.06, 0.58)	0.015	0.23 (-0.04, 0.51)	0.093
**Model 6:**								
None	ref		ref		ref		ref	
Emotional (2 or more)	0.46 (0.28, 0.64)	<0.001	0.35 (0.17, 0.53)	<0.001	0.31 (0.03, 0.59)	0.033	0.14 (-0.15, 0.42)	0.342
Emotional (2 or more) and p/s (2 or more)	0.83 (0.72, 0.94)	<0.001	0.48 (0.36, 0.60)	<0.001	1.05 (0.82, 1.28)	<0.001	0.61 (0.37, 0.86)	<0.001
Physical and/or sexual only (2 or more)	0.51 (0.36, 0.66)	<0.001	0.41 (0.27, 0.54)	<0.001	0.51 (0.20, 0.82)	0.002	0.38 (0.06, 0.71)	0.02
**Model 7:**								
None	ref		ref		ref		ref	
Economic only (1 or more)	0.25 (0.06, 0.44)	0.009	0.12 (-0.07, 0.32)	0.203	0.41 (0.05, 0.78)	0.028	0.30 (-0.03, 0.64)	0.078
Economic (1 or more) and p/s (1 or more)	0.84 (0.70, 0.97)	<0.001	0.49 (0.34, 0.63)	<0.001	1.10 (0.88, 1.32)	<0.001	0.70 (0.49, 0.92)	<0.001
Physical and/or sexual only (1 or more)	0.45 (0.34, 0.57)	<0.001	0.30 (0.19, 0.42)	<0.001	0.49 (0.27, 0.72)	<0.001	0.34 (0.11, 0.57)	0.005
**Model 8:**								
None	ref		ref		ref		ref	
Economic (2 or more)	0.45 (0.27, 0.62)	<0.001	0.28 (0.11, 0.46)	0.002	0.62 (0.26, 0.99)	0.001	0.46 (0.12, 0.80)	0.008
Economic (2 or more) and p/s (2 or more)	0.87 (0.75, 0.98)	<0.001	0.50 (0.36, 0.64)	<0.001	1.21 (0.95, 1.46)	<0.001	0.79 (0.53, 1.05)	<0.001
Physical and/or sexual only (2 or more)	0.57 (0.46, 0.68)	<0.001	0.37 (0.27, 0.48)	<0.001	0.67 (0.40, 0.94)	<0.001	0.46 (0.18, 0.73)	0.001

*Models 5-8: Different IPV combinations experienced by women, disregarding IPV experience not mentioned.

†Negative binomial regression.

‡Ordered logistic regression.

§Adjusted for: age, education, poverty and childhood traumas.

**Table 5:** Adjusted and unadjusted associations between depression and IPV experience, and self-reported health and IPV experience*

	Depression*				Self-rated health†			
	**Unadjusted**		**Adjusted‡**		**Unadjusted**		**Adjusted‡**	
	**β (95% CI)**	***P*-value**	**β (95% CI)**	***P*-value**	**β (95% CI)**	***P*-value**	**β (95% CI)**	***P*-value**
**Model 9:**								
None	ref		ref		ref		ref	
Emotional only	0.24 (0.05, 0.43)	0.016	0.21 (0.02, 0.40)	0.032	0.370.07, 0.66)	0.017	0.26 (-0.05, 0.56)	0.1
Economic only	0.05 (-0.23, 0.33)	0.724	-0.05 (-0.29, 0.19)	0.676	0.31 (-0.06, 0.68)	0.103	0.26 (-0.09, 0.61)	0.147
Emotional and economic only	0.62 (0.36, 0.88)	<0.001	0.46 (0.19, 0.73)	0.001	0.70 (0.12, 1.28)	0.018	0.49 (-0.01, 1.00)	0.056
Emotional and physical/sexual only	0.61 (0.48, 0.73)	<0.001	0.41 (0.29, 0.52)	<0.001	0.71 (0.41, 1.02)	<0.001	0.49 (0.17, 0.80)	0.003
Economic and physical/sexual only	0.59 (0.33, 0.85)	<0.001	0.44 (0.19, 0.70)	0.001	0.58 (0.15, 1.01)	0.009	0.47 (0.02, 0.92)	0.04
Physical/Sexual only	0.31 (0.10, 0.52)	0.004	0.26 (0.06, 0.46)	0.012	0.29 (0.00, 0.59)	0.053	0.22 (-0.10, 0.53)	0.18
All IPV experienced	0.92 (0.78, 1.06)	<0.001	0.55 (0.40, 0.70)	<0.001	1.24 (1.00, 1.47)	<0.001	0.80 (0.56, 1.04)	<0.001
**Model 10:**								
None	ref		ref		ref		ref	
Emotional only (2 or more)	0.30 (0.09, 0.51)	0.006	0.26 (0.04, 0.49)	0.021	0.17 (-0.13, 0.46)	0.264	0.07 (-0.23, 0.38)	0.635
Economic only (2 or more)	0.24 (-0.02, 0.49)	0.06	0.15 (-0.08, 0.39)	0.201	0.50 (0.11, 0.89)	0.013	0.42 (0.05, 0.80)	0.026
Emotional (2 or more) and economic (2 or more)	0.84 (0.60, 0.86)	<0.001	0.59 (0.35, 0.83)	<0.001	0.86 (0.34, 1.39)	0.001	0.54 (0.05, 1.03)	0.03
Emotional (2 or more) and physical/sexual (2 or more)	0.72 (0.59, 0.86)	<0.001	0.47 (0.33, 0.83)	<0.001	0.82 (0.49, 1.15)	<0.001	0.51 (0.17, 0.86)	0.004
Economic (2 or more) and physical/sexual (2 or more)	0.77 (0.50, 1.04)	<0.001	0.64 (0.37, 0.90)	<0.001	0.76 (0.26, 1.27)	0.003	0.66 (0.16, 1.17)	0.01
Physical/Sexual only (2 or more)	0.42 (0.25, 0.59)	<0.001	0.34 (0.17, 0.51)	<0.001	0.49 (0.13, 0.85)	0.008	0.38 (0.00, 0.76)	0.051
All IPV experienced (2 or more)	0.93 (0.81, 1.05)	<0.001	0.54 (0.40, 0.69)	<0.001	1.28 (1.01, 1.56)	<0.001	0.82 (0.53, 1.11)	<0.001

*Negative binomial regression.

†Ordered logistic regression.

‡Adjusted for: age, education, poverty and childhood traumas.

For economic IPV, independent associations with worse health outcomes are stronger for self-reported health, particularly when coded as two or more experiences. In descriptive models, and adjusted models, when economic IPV is coded as two or more there are consistent associations with worse self-rated health, with no overlap of 95% CIs in descriptive models (Table S3 in the **Online Supplementary Document**), and in all adjusted models (**Table 4**, Model 8; **Table 5**, Model 10). There are less consistent findings when economic IPV is treated as one or more experiences. Similarly, there are more mixed findings for economic IPV and its association with depressive symptoms, where the models show inconsistent findings.

Across all models women who experience physical and/or sexual IPV, when combined with emotional or economic IPV consistently strong associations with worse health outcomes, and the highest scores for depression and self-rated health. For instance in Table S3 in the **Online Supplementary Document****,** all combinations of combined IPV show the highest mean scores for symptoms. Similarly, in all the adjusted regression models, combined experiences of IPV are show the largest coefficients for depressive symptoms and self-rated health, and are in all models these associations are highly significant (**Table 4** and **Table 5**).

## DISCUSSION

In three population-based samples from the Asia-Pacific region (Sri Lanka, China, and PNG), lifetime experiences of emotional IPV and economic IPV were common for women. Emotional IPV was the most prevalent form of lifetime IPV assessed ranging from 29.5% in Sri Lanka, 37.7% in China, to 69.0% in PNG. Lifetime prevalence of economic IPV was also high, ranging from 24.7% in China, 27.0% in Sri Lanka, to 55.4% in PNG. The comparative prevalence of emotional IPV and economic IPV tracked closely with the prevalence of IPV by country with participants in PNG reporting the highest prevalence of physical IPV and sexual IPV, as well as emotional IPV, and economic IPV.

While emotional IPV and economic IPV were common across all three countries, there were differences in the most common items reported for each type of IPV by country. For emotional IPV, “scaring and intimidating” was the most common item in Sri Lanka and China, while in PNG it was “insulted”. There was little consistency in terms of ‘uniqueness’ of items by country. Only one item, ‘hurt others,’ had low uniqueness across the three countries, with <3% of women reporting emotional IPV reporting only that item. There was also variation in most prevalent form of economic IPV by country. In Sri Lanka and China ‘prohibit from working’ was the most common, while in PNG it was ‘taken earnings’. There was no item from the economic IPV scale, which had consistently low uniqueness.

The variation in the most common items of emotional and economic IPV and limited set of items with consistently low levels of uniqueness across the three countries has two implications. First, the variation in reporting across different countries suggests that while both emotional IPV and economic IPV are commonly experienced, the forms they take vary by context, and this is likely linked to different norms around relationships, women’s mobility, the expectations around women’s work, and male provision in relationships [9], as well as the nature and patterning of poverty. For example formal work in PNG was relatively uncommon (21% in this study), compared to the Sri Lanka and China where women’s employment is more common [21,22]. Similarly, men refusing to give women money when there was enough is also highly contingent on how the distribution of economic resources are understood in relationships.

Second, the current set of commonly used emotional IPV and economic IPV indicators cannot be easily reduced, as there were very items that across all countries had a low level of uniqueness. The only item that could potentially be removed to shorten questionnaires for emotional IPV was “hurt others” where <3% of women only reported that form of emotional IPV. If removed, in these three populations, the prevalence of emotional IPV would be about 3% less. As such, this suggests the current set of items do provide a unique set of items that do not repeat one another.

Our analysis clearly demonstrates that emotional IPV and economic IPV have negative health impacts on women, and that this impact is independent of the impact of co-occurring physical and/or sexual IPV. This was demonstrated across a range of different modelling and coding strategies, with consistent patterning of findings. Importantly, given the large data set we were able to parse out the multiple different combinations of IPV experienced by women, thus limiting the potential for hidden cofounders of IPV experience.

There was also greater consistency for the health impacts of emotional IPV and economic IPV (when excluding physical and/or sexual IPV), when these constructs were coded as “never or once” vs “two or more” experiences, rather than a “never/ever” binary. This suggests that coding emotional and economic IPV as none or once vs two or more, may be the appropriate way to capture a level of IPV where negative health impacts are seen.

The analysis suggests that while capturing emotional IPV and economic IPV in terms of frequency of reporting (never, once, few, many), may be useful in looking at severity, in reality emotional IPV and economic IPV functioned best as binary measures. When examining frequency and health impacts, there were inconsistencies in reporting, with some women reporting worse health outcomes for ‘few’ rather than ‘many’ experiences. The majority of women reporting any emotional IPV or economic IPV experienced this multiple times, rather than just once, and it may be that the response of never, once, few, or many, categories do not resonate with women’s experiences of these forms of violence, which tend not to be discrete experiences. As such, developing a range of responses that resonate more closely with women’s experiences may be an important future step.

Where women experienced multiple forms of IPV, ie, emotional and economic combined with physical and/or sexual IPV, they reported the worst health outcomes, and this is likely an indication of the severity of IPV experienced. This is the same finding as Gibbs et al [11] found in a non-representative sample in South African young women. Currently there is no agreed upon approach as to how to assess the severity of IPV, with some approaches suggesting two or more experiences of physical/sexual IPV [23], while others suggesting a more complex coding based on specific acts women experience [24]. This coding of women who experience physical and/or sexual IPV as well as emotional or economic IPV, may be a simple way of modelling severity, which is robust in population data.

Emotional IPV was consistently associated with depressive symptoms, but not self-rated generalised health, while economic IPV was more consistently associated with worse self-rated generalised health. This patterning was seen in both the individual items of each scale, and in the combined violence measures where different experiences were parsed out. It is unclear why these specific associations were seen, although the few other studies looking at both forms of IPV, have also demonstrated differences in health impacts [25]. This is suggestive that emotional IPV and economic IPV may operate somewhat differently and further research is required to understand this.

This study has a number of limitations. The measures of emotional IPV and economic IPV were for lifetime occurrence, while depressive symptoms were past week, and self-reported health the current period. As such, reports of IPV may have been from a long time prior to the survey. Despite this lack of congruence of reporting periods the pronounced impact on women’s health was still identified, which may be because many women experience IPV repeatedly, rather than just once-off. We only assessed depressive symptoms, rather than providing a clinical diagnosis and generalized health was a single item, and as such, the study needs repeating with more robust health outcomes. We did not create entirely “clean” referent categories for all analyses due to sample size issues, so in some cases a small proportion may have reported either emotional IPV or economic IPV, rather than truly “none”. The data were population representative samples and as such are generalizable to the wider population they came from, but the population was not always the whole country.

## CONCLUSIONS

In conclusion, emotional IPV and economic IPV are more common, or as common as physical IPV and sexual IPV in three countries in Asia-Pacific. This analysis has highlighted that the current set of emotional and economic items used by the WHO captures a range of unique instances of violence that women face from their partner and that forms of emotional and economic IPV are patterned across different contexts. However the item list is unlikely to be exhaustive and future research may want to explore if important additional items need to be added for particular contexts. In addition, it has demonstrated that simple binary coding of these scales provides a robust way of providing a measure of health impact. The simplicity of this approach enables replication and standardization of measurement of these key constructs across multiple settings, enabling comparison.

## Additional material

Online Supplementary Document
